# Development of Eco-Friendly Silane-Treated Rice Flour/PBS Biocomposites with ENR-50 as a Compatibilizer: A Study on Phase Morphology, Properties and Biodegradation

**DOI:** 10.3390/polym17162213

**Published:** 2025-08-13

**Authors:** Thritima Sritapunya, Apaipan Rattanapan, Surakit Tuampoemsab, Pornsri Sapsrithong

**Affiliations:** Division of Polymer Engineering and Rubber Industrial Technology, Department of Mechanical Engineering Technology, College of Industrial Technology, King Mongkut’s University of Technology North Bangkok, Bangkok 10800, Thailand; thritima.s@cit.kmutnb.ac.th (T.S.); apaipan.r@cit.kmutnb.ac.th (A.R.); surakit.t@cit.kmutnb.ac.th (S.T.)

**Keywords:** PBS-based biocomposites, sustainable materials, agro-waste filler, rice flour filler, silane treatment, epoxidized natural rubber (ENR), mechanical performance, soil burial degradation, renewable resources

## Abstract

This study investigated the development of biocomposites for use as packaging and film in everyday applications. The utilization of rice flour (RF) as a cheap natural filler in the production of polybutylene succinate (PBS) biocomposites has been shown to reduce environmental issues caused by non-biodegradable plastic waste. The effect of rice flour content on the morphology and properties of PBS and RF biocomposites was comprehensively evaluated. Different amounts of rice flour were considered (0, 10, 20, 30, 40, and 50 phr), and a silane coupling agent and epoxidized natural rubber (ENR-50: 1 phr) were used as interfacial agents to improve compatibility between the matrix (PBS) and filler (RF). The PBS/RF biocomposites were prepared using a two-roll mill and shaped into test specimens and films using a compression molding machine. Batches of the composites containing different amounts of RF were prepared in accordance with the standards, and their morphology and properties, including mechanical properties, density, water absorption, and soil burial degradation, were evaluated. The results revealed that the incorporation of silane-treated RF filler and ENR-50 compatibilizer led to notable improvements in mechanical properties, particularly in tensile modulus, flexural strength, flexural modulus, and hardness. A significant improvement in mechanical performance was observed as the RF content increased, with the highest value recorded at the 50 phr loading. The enhancements observed in the composite properties are due to the inherent rigidity of the RF filler and its improved compatibility with the PBS matrix, which together contribute to a stronger and more efficient material. Additionally, the percentage of water absorption in the PBS/RF biocomposites increased with higher RF content. The results from the soil burial test demonstrated that increasing the RF content positively influenced the biodegradability of the PBS/RF biocomposite materials.

## 1. Introduction

The extensive use of non-biodegradable plastics made from raw materials derived from petroleum has become a major source of environmental waste pollution. These plastics take a long time to break down and decompose. Plastics pose serious threats to the environment and to people when they are burned, as they increase air pollution and the greenhouse effect. Furthermore, non-biodegradable plastics disintegrate into microplastics over time, risking the health of living species and disrupting ecosystems. As a result, attempts to reduce global plastic usage have been boosted by growing public awareness of these issues. Efforts to promote environmentally friendly packaging materials, including fabric-based and biodegradable materials, have gained momentum; however, habitual dependence on plastic persists, driven by culturally embedded usage patterns and resistance to behavioral change.

Bio-based polymers made from renewable resources have become increasingly popular in recent years. Polybutylene succinate (PBS) and polylactic acid (PLA) are two famous bio-based plastics that have gained worldwide attention [1,2]. Consequently, ongoing research and innovation have prioritized the development of bio-based plastics that align with environmental sustainability goals as well as cater to the demands of contemporary urban living. After undergoing microbial and enzymatic degradation, biodegradable plastics—a subset of environmentally responsive materials—break down into carbon dioxide, methane, water, and biomass—byproducts that reintegrate into natural ecosystems and significantly lessen the long-term ecological burden compared to petroleum-based plastics. Polybutylene succinate (PBS) is recognized for its versatile properties, including robust mechanical strength, superior flexibility, and thermal stability that allows it to maintain structural integrity at elevated temperatures [2,3]. Additionally, the excellent processability of PBS also enables efficient fabrication into a wide range of end-use products. Although PBS offers excellent thermal and mechanical characteristics, its production cost is higher than conventional petroleum-based plastics, hindering large-scale commercialization. The degradation rate of PBS is also relatively slow under ambient conditions, often requiring industrial composting environments to achieve effective breakdown. Therefore, to overcome these limitations, recent studies have focused on blending PBS with other biodegradable polymers or reinforcing it with natural fibers to improve both mechanical strength and the biodegradation rate under ambient conditions, as well as cost reduction.

The utilization of natural fillers such as peanut shells, bamboo, flax, pineapple leaf, jute, sisal fiber, bagasse fiber, rice husk, rice straw, and rice flour—derived from agricultural residues—offers a promising approach for the development of biodegradable materials with desirable properties such as excellent mechanical properties, non-toxicity and low costs [3,4,5,6,7,8,9,10,11]. Bio-based polymer composites offer a promising alternative to conventional non-biodegradable composites, with the potential to reduce the environmental impact and support sustainable material development. According to the study by Mayank Pokhriyal and Pawan Kumar Rakesh (2024), the incorporation of natural fillers significantly improved the mechanical properties of polylactic acid (PLA) biocomposites reinforced with *Himalayacalamus falconeri* fibers [6]. Additionally, the use of *Himalayacalamus falconeri* (a type of bamboo) fiber as a bio-reinforcement in the PLA matrix has also been explored. Mechanical performance, including tensile, flexural, and impact strengths, showed notable enhancement with filler loading up to 15 wt.%. Biodegradability was assessed through soil burial testing by measuring the percentage of weight loss before and after exposure. Studies have demonstrated that the presence of bio-based fiber in the PLA matrix accelerates the degradation rate under soil burial conditions, with the material properties and extent of degradation being significantly affected by the amount of filler content in the polymer matrix [12]. The utilization of rice waste such as husk, straw, bran, and flour as a reinforcing component in polymer composites has emerged as a strategic direction in advancing eco-friendly, low-cost, and sustainable composite technologies [10,11]. Recent studies have investigated biocomposite films composed of polylactic acid (PLA), natural rubber (NR), and rice straw (RS), aiming to enhance the sustainability and mechanical performance of biodegradable materials. The addition of natural components accelerated the biodegradation rate of the composites under soil burial conditions [9].

Rice flour is a starch-rich powder produced through the milling of rice grains, often sourced from broken rice or residuals of rice processing. The primary component of rice flour is starch, with trace amounts of proteins, lipids, and minerals [13,14]. As a sustainable filler in biodegradable polymer composites, rice flour has received much attention because of its natural availability, renewability, and strong compatibility with biopolymer matrices. Sarekha Woranuch et al. (2017) studied the influence of rice flour content on the viscosity, morphology, chemical structure, crystallinity, thermal characteristics, and mechanical properties of rice flour/polyvinyl alcohol (PVA) blended nanofibers [13]. The incorporation of rice flour into the PVA solution resulted in enhanced tensile strength and Young’s modulus compared to neat PVA nanofibers, suggesting improved mechanical reinforcement through filler–matrix interaction. Enhancing the characteristics of the composite requires improving the interfacial adhesion between the matrix and the fiber or filler. This can be effectively achieved through silane surface modification and the use of compatibilizers like maleic anhydride grafted polymers and epoxy functional compatibilizers, which contribute to better dispersion and interfacial bonding [15,16,17,18,19,20,21,22].

The compatibilizing ability of ENR-50 has been established in various polymer systems, including LLDPE/soya powder blends [18], poly(lactic acid)/rice starch composites [19], and PA6/PBT blends [20]. They reported that the improvement in morphology and mechanical properties was observed, and a small amount of ENR-50 is adequate to act as an effective compatibilizer in enhancing interfacial interactions within polymer blends and composites. Sam et al. investigated the role of epoxidized natural rubber (ENR-50) as an effective compatibilizer in improving the blend compatibility between linear low-density polyethylene (LLDPE) and soya powder [18]. The improvement in morphology and tensile properties was investigated with the addition of ENR-50 as a compatibilizer because of the enhanced interfacial adhesion between LLDPE and soya powder. Yew et al. [19] investigated the effect of ENR-50 as a compatibilizer in PLA/rice starch composites. Incorporating a small amount of ENR-50 as a compatibilizer significantly enhanced the mechanical properties by effectively improving the interfacial adhesion between the components. However, excessive amounts of ENR-50 may adversely affect certain properties of the polymer composite and can lead to phase separation and reduced interfacial adhesion, ultimately resulting in a decline in some mechanical or physical properties. This decline could result from the elastomeric properties and the compatibilizing action of ENR-50. Moreover, it has been reported that the use of dual compatibilizers performs better than a single compatibilizer in optimizing composite properties [21,22]. Therefore, this study employs a combination of a silane coupling agent and ENR-50 as interfacial agents to enhance both the compatibility and overall performance of the polymer composite.

This study investigated biocomposites made of rice flour (RF) filled with PBS, and the effects of RF concentration on phase morphology, mechanical properties (tensile properties, flexural properties, and hardness), density, water absorption, and soil burial degradation were studied. In addition, interfacial compatibility between the PBS matrix and the RF filler was enhanced through the surface modification of the RF filler using silane treatment, along with the incorporation of 1 phr of epoxidized natural rubber (ENR-50) as a compatibilizing agent.

## 2. Materials and Methods

### 2.1. Materials

Poly(butylene succinate) (PBS: BioPBS™ FD92PM Lot. TN0P28B No.0395 for film packaging application), with a density of 1240 Kg/m^3^, was used as the polymer matrix, and it was supplied by PTT MCC BIOCHEM Co., Ltd. (Bangkok, Thailand). The broken rice (rice flour: RF) used in this study was collected from a local factory in Thailand’s Phichit province (Phichit, Thailand), ground to powder, and screened through laboratory sieves (mesh: 100). The rice flour was used as a filler in the PBS biocomposite. The epoxidized natural rubber contained approximately 50 mol% epoxide groups (ENR-50), supplied by Muang Mai Guthrie Public Co., Ltd. (Bangkok, Thailand), and was employed as a compatibilizer. To enhance interfacial adhesion between the PBS matrix and rice flour, 3 wt.% solution of the silane coupling agent (3-aminopropyl-triethoxysilane: Dynasylan^®^ AMEO, Evonik Degussa GmbH, Essen, Germany) was applied, and the filler was subsequently immersed in a 3 wt.% silane solution in ethanol for 30 min. Following treatment, the RF was dried for 24 h at 60 °C in a hot-air oven to eliminate any remaining solvents.

### 2.2. Compounding and Specimen Preparation

Rice flour was treated with a silane coupling agent, and 1 part per hundred resin (phr) of epoxidized natural rubber (ENR-50) was utilized as a compatibilizer to increase compatibility between PBS (matrix) and rice flour (filler). Different amounts of rice flour—0, 10, 20, 30, 40, and 50 phr—were used to prepare PBS/RF biocomposites (Table 1). Prior to melt blending, PBS and rice flour were dried in an oven at 60 °C for 24 h to minimize moisture content. The materials were blended in a two-roll mill (LRM-S-110/3E, Labtech Engineering Co., Ltd., Samutprakarn, Thailand) at 155 °C for 10 min. The PBS/RF biocomposites were allowed to dry at room temperature before being granulated and shaped into test specimens using a compression molding machine (LP20-B, Labtech Engineering Co., Ltd., Samutprakarn, Thailand) at 155 °C for 5 min, followed by cooling for 5 min. All molded specimens and films were conditioned at 25 ± 2 °C and 50 ± 5% relative humidity for a minimum of 48 h to ensure stabilization of physical properties prior to characterization. After this conditioning step, the samples underwent comprehensive characterization.

### 2.3. Characterizations

FTIR spectroscopy (Thermo Scientific/Nicolet Nexus IS5, Thermo Fisher Scientific Inc., Waltham, MA, USA) equipped with an Attenuated Total Reflectance (ATR) accessory was employed to examine the chemical interactions between rice flour and the silane coupling agent. Spectra were collected in transmittance mode over a broad wavenumber range of 650–4000 cm^−1^, at a resolution of 4.0 cm^−1^, to reveal detailed insights into molecular bonding and interfacial phenomena within the samples.

The density and water absorption of PBS/RF biocomposites were determined according to ASTM D792-13 (2013) [23] and D570-22 (2022) [24], respectively. Using a Sartorius balance (Model MDS-300, ALFA MIRAGE Co., Ltd., Osaka, Japan) and the Archimedean principle (water displacement method), the density of the composites was determined. As the liquid, distilled water was utilized. An average of three measurements was provided for every sample. Samples were subjected to weighing in ambient conditions and again while submerged in distilled water to determine their apparent mass. An analytical balance was used to ensure accurate measurement, and density was calculated in accordance with ASTM D792. For water absorption, the samples were submerged in distilled water for 24, 48, 72, 120, 168, and 360 h at room temperature in order to assess the water absorption of composites. At each interval, specimens were removed, lightly wiped with a facial tissue to eliminate excess surface moisture, and weighed promptly using a high-accuracy balance. The percentage of water absorption was calculated based on the weight difference in the specimens before and after immersion, using the initial dry weight as a reference. Tensile testing was carried out in accordance with ASTM D882-18 (2018) [25] to determine the tensile stress and Young’s modulus of the composite films. A universal testing machine (M500-25AT, Testometric Co., Ltd., Rochdale, UK) was used for the measurement, equipped with a load cell of 100 kgf, using a crosshead speed of 100 mm/min. The flexural strength and modulus were determined following ASTM D790-15 (2015) [26]. Rectangular samples were tested under the three-point bending mode at a crosshead speed of 1.30 mm/min using a universal testing machine. At least three samples were tested for each evaluation to ensure accuracy and consistency. A hardness shore durometer (Type shore D, DESIK, Germany) was used to measure hardness in accordance with the ASTM D2240-15 (2015) [27] test protocol. The indentation resistance of the molded specimens was quantified using the Shore D scale. Measurements were averaged from ten different locations to account for surface variation.

Phase morphological characterization was performed on the test specimens using a scanning electron microscope (FE-SEM, JEOL, Hitachi, Model S4800, Tokyo, Japan). A field emission scanning electron microscope (FE-SEM), operated at an accelerating voltage of 10 kV, was employed to examine the surface morphology of the fractured composite specimens. Prior to imaging, the samples were sputter-coated with a thin gold layer to enhance conductivity and minimize charging effects. Elemental analysis was carried out on the fractured surface of the PBS/RF biocomposites using energy-dispersive X-ray spectroscopy (EDX) integrated with the FE-SEM system under the same operating voltage.

The degradation rate of PBS/RF biocomposites was determined using a soil burial degradation test. The samples were retrieved after being buried for periods of 30, 60, 90, 180, and 270 days. In a plastic container, rectangular test specimens (1 cm × 5 cm × 0.3 cm) and thin film samples (10 mm × 130 mm × 0.03 mm) were buried in natural soil at a depth of 8 cm under ambient outdoor conditions, enzyme-free soil without any compost additives, to evaluate their biodegradability under ambient conditions. After the buried samples were collected at a specific time, they were carefully washed to get rid of any remaining dirt residue and allowed to air-dry in a hot-air oven at a temperature of 50 °C until their mass remained consistent. Weight loss was calculated from the weight of the sample before and after testing at specific times and evaluated using Equation (1). In addition, FE-SEM was used for visual comparison to determine the fractured surface appearances of the soil-buried samples.

Weight Loss (%) = [W_0_ − W_1_/W_0_] × 100 (1)

where W_0_ and W_1_ are the weights of the sample before and after the soil burial test, respectively.

## 3. Results and Discussion

### 3.1. FTIR Analysis of Rice Flour

**Figure 1:**
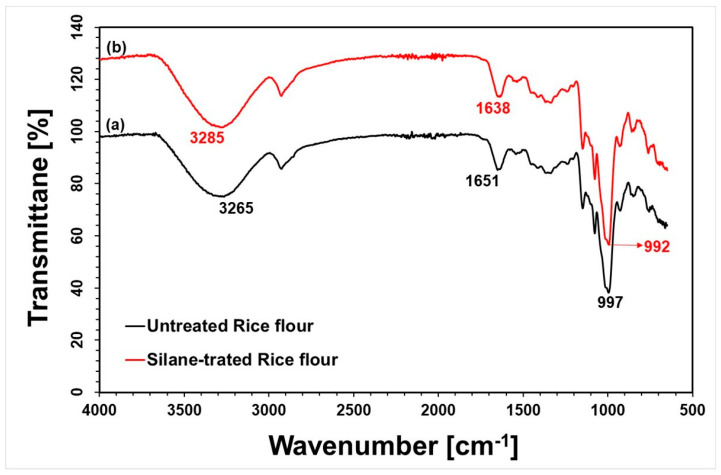
FTIR spectra of rice flour: (a) Untreated rice flour and (b) Silane-treated rice flour.

Fourier-transform infrared (FTIR) spectroscopy was utilized to investigate the interactions between functional groups and to assess the compatibility at the interface between rice flour filler and silane coupling agents. Figure 1 shows the FTIR spectra of (a) untreated rice flour (RF) and (b) silane-treated rice flour. The spectrum of untreated rice flour prominently features vibrational bands corresponding to O–H, C–H, C=O, and C–O functional groups, reflecting its complex composition of polysaccharides (primarily starch) and proteins [28]. A distinct absorption band near 1651 cm^−1^ is attributed to the C=O stretching vibrations in the amide I region of proteins, while the broad band at approximately 3265 cm^−1^ corresponds to O–H stretching vibrations from hydroxyl groups in rice flour. Both untreated and silane-treated rice flour spectra show characteristic peaks at similar wavenumbers; however, notable spectral changes after silane treatment indicate successful chemical modification [29]. These changes include slight shifts and increased intensity in absorption bands. Specifically, the O–H peak shifts from 3265 to 3285 cm^−1^, and the C=O peak shifts from 1651 to 1638 cm^−1^, with increased intensities at these positions and near 990–1000 cm^−1^. The enhanced absorption around 990–1000 cm^−1^ is characteristic of siloxane-related stretching vibrations (Si–O–Si and Si–O–C bonds), strongly indicating that silane molecules have chemically bonded to the rice flour surface, forming siloxane linkages. This evidence confirms successful compatibilization and chemical interactions between the rice flour filler and the silane coupling agent [30].

### 3.2. Morphology

**Figure 2:**
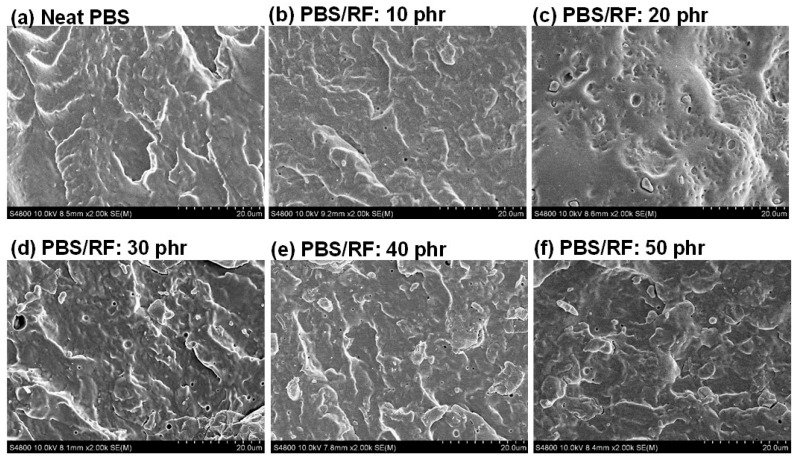
SEM micrographs of PBS/RF biocomposites.

The influence of rice flour (RF) content on the phase morphology and characteristics of polymer composites made from PBS (matrix) and RF (filler) was investigated. The distribution and phase morphology of RF in the PBS matrix were determined using FE-SEM. The incorporation of RF filler resulted in a well-dispersed phase within the matrix, without the formation of noticeable agglomerates. SEM images of the PBS/RF biocomposites with various amounts of silane-treated rice flour and 1 phr of ENR-50 as a compatibilizer (Figure 2) showed no evidence of phase separation between the PBS matrix and RF filler. However, a distinct increase in surface roughness, compared to that of the neat PBS, was observed when the filler content reached 50 phr. The fractured surface roughness of the PBS/RF biocomposites exhibited a noticeable increase as the RF content increased from 0 to 50 phr, suggesting enhanced mechanical interlocking between the filler and the matrix. The presence of silane coupling agent and ENR-50, acting as interfacial agents, enhances the interfacial compatibility between PBS and RF. Consequently, the biocomposites demonstrated superior tensile modulus, flexural strength, and flexural modulus compared to the neat PBS [15,17,18].

**Figure 3:**
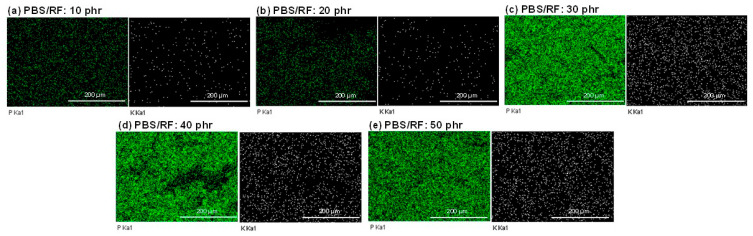
P and K mapping via energy-dispersive X-ray (EDX) analysis of PBS/RF biocomposites.

To investigate the influence of filler content on the microstructure of the polymer composites, scanning electron microscopy (SEM) coupled with energy dispersive X-ray spectroscopy (EDX) was employed to analyze the morphology and elemental distribution within the samples. EDX analysis of the PBS/RF biocomposites was also conducted to confirm the presence of major elements, including potassium (K) and phosphorus (P), the principal elements in rice flour, and to evaluate the distribution of these fillers within PBS/RF biocomposites. In Figure 3, elemental mapping via EDX demonstrated a homogeneous distribution of rice flour particles, with sporadic appearances of potassium and phosphorus mineral elements. Compared to the neat PBS, the rice flour-filled composites exhibited an increase in inorganic element signals, notably potassium and phosphorus. The amount of K and P increased with the amount of added rice flour up to 50 phr. Furthermore, the EDX elemental mapping demonstrated a uniform dispersion of potassium (K) and phosphorus (P) within the PBS/RF composite matrix.

### 3.3. Physical and Mechanical Properties

**Figure 4:**
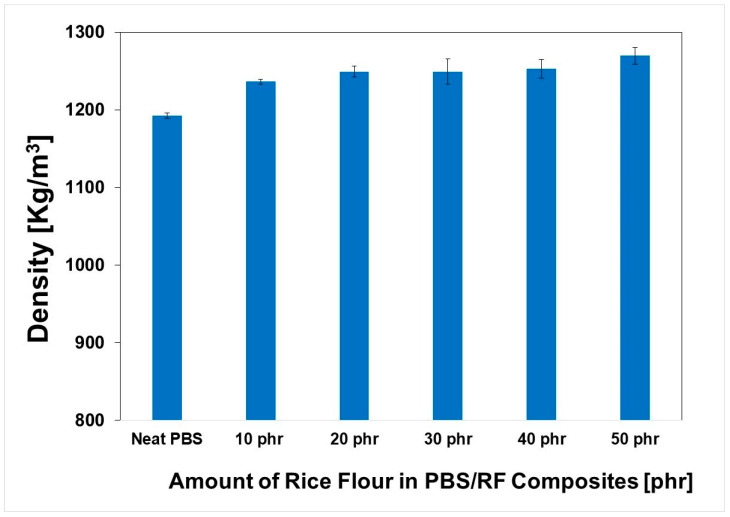
Density of PBS/RF biocomposites.

The apparent density (Kg/m^3^) of the PBS/RF biocomposites was evaluated using the water immersion method, according to ASTM D792 (see Figure 4). The weight of each specimen was recorded in air, and its apparent volume was measured when fully submerged in water. The densities of the composites depended on the amount of RF filler loading in PBS, with the density of RF filler higher than that of the PBS matrix, as well as on the satisfactory dispersion and distribution of RF filler in the polymer matrix, which was in agreement with the literature [31,32]. Aung Kyaw Moe et al. (2022) demonstrated that adding NMPCB to a PVC matrix resulted in a significant increase in PVC/NMPCB composite density. This increase correlated with the NMPCB content rising from 10 to 30 wt.%, which is likely due to the relatively higher density of NMPCB filler compared to the PVC matrix [31]. Therefore, adding natural filler to the PBS matrix increased the density of the PBS/RF biocomposites from 1192.3 for neat PBS to 1269.7 Kg/m^3^ for the PBS/RF biocomposite (50 phr).

**Figure 5:**
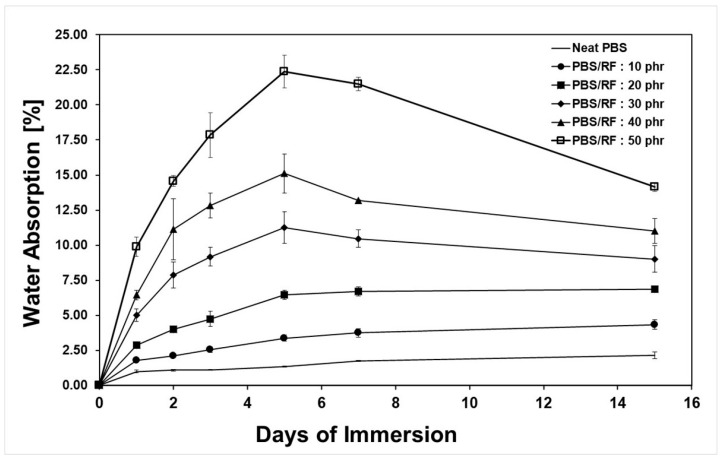
Water absorption of PBS/RF biocomposites.

The PBS/RF biocomposite specimens were tested for water absorption according to ASTM D570-22 (2022). Figure 5 presents an evaluation of the percentage of water absorption of the PBS/RF biocomposites at various testing times when submerged in distilled water at room temperature. It was noted that the ability of the PBS/RF biocomposites to absorb water increased as the amount of RF filler increased, with the neat PBS absorbing the least water. After 24 h, the composite with 50 phr RF filler absorbed the most water, showing an 889% increase compared to the neat PBS, probably because the RF filler attracted water and created a higher surface area in the composite, allowing for more water to be absorbed over time. This increase was probably caused by the hydrophilic nature of the RF filler and the larger interfacial area within the matrix, and the composite continued to absorb more water as the test duration increased [17,33]. Thus, the comparatively high water-absorption characteristic of the natural filler played a role in the increased water absorption observed in the composites. Kampeerapappun et al. (2024) [17] observed similar results in their study of PLA/rice straw (RS) composites. They used rice straw as a natural filler, which is a hydrophilic material, to improve the percentage of water absorption of the PLA/rice straw (RS) composites. The reduced water absorption observed in high-filler composites (30 to 50 phr) after prolonged immersion is attributed to the partial degradation or leaching of the filler phase, leading to structural breakdown and subsequent detachment of loosely bound particles from the matrix. This detachment causes a loss of mass in the composite sample, which consequently contributes to a decrease in the measured water absorption values. This observation is consistent with the soil burial degradation test results, which revealed that the degradation rate was influenced by the filler content; as the RF filler loading increased, the composite underwent more rapid degradation over time [1,2,12]. Similar observations have been reported by Then et al. (2015). They studied biocomposites made from poly(butylene succinate) (PBS) reinforced with varying contents of oil palm mesocarp fiber (OPMF). Their findings demonstrated that PBS/OPMF biocomposites showed improved water uptake and biodegradability compared to neat PBS, and that these properties increased with fiber content [12].

**Figure 6:**
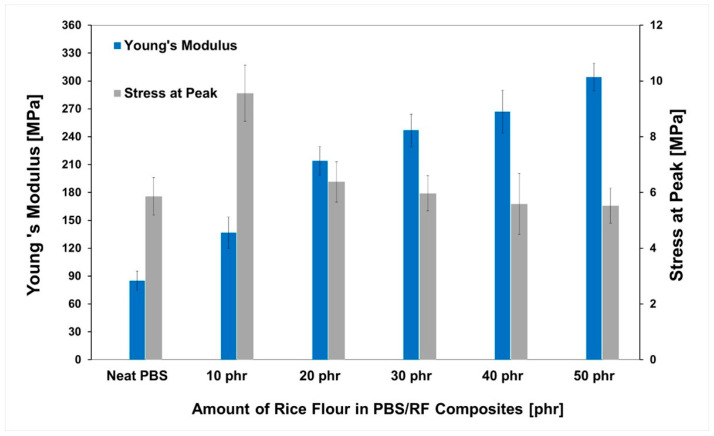
Tensile properties of PBS/RF biocomposites.

PBS/RF biocomposites were prepared via melt compounding using silane-treated rice flour (RF) and 1 phr of epoxidized natural rubber (ENR-50) and shaped into films and specimens for mechanical testing. In Figure 6, the Young’s modulus and the stress at the peak of the resulting composite films are presented as a function of RF content ranging from 0 to 50 phr. An increasing trend in Young’s modulus was observed with higher RF loading compared to the neat PBS. This enhancement is attributed to the greater stiffness of RF particles relative to the PBS matrix, which contributed to the improved rigidity of the composites [31]. Furthermore, silane surface treatment and the use of ENR-50 compatibilizer promoted interfacial interactions, which contributed to a uniform dispersion of the RF filler within the PBS matrix. The stress at peak values of the PBS/RF biocomposite film as a function of RF content was also measured (Figure 6). Maximum improvement was observed with the addition of only 10 phr of RF into the PBS matrix. Although the filler was well dispersed throughout the matrix, the tensile stress decreased due to increased material brittleness and limited polymer chain mobility caused by the presence of rigid filler particles.

**Figure 7:**
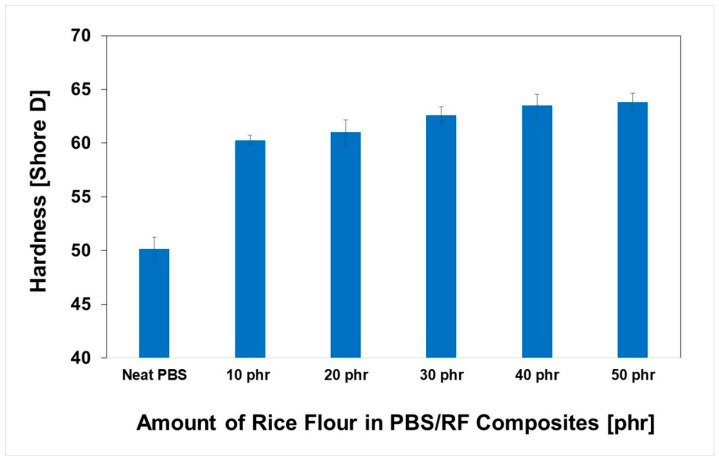
Hardness (Shore D) of PBS/RF biocomposites.

**Figure 8:**
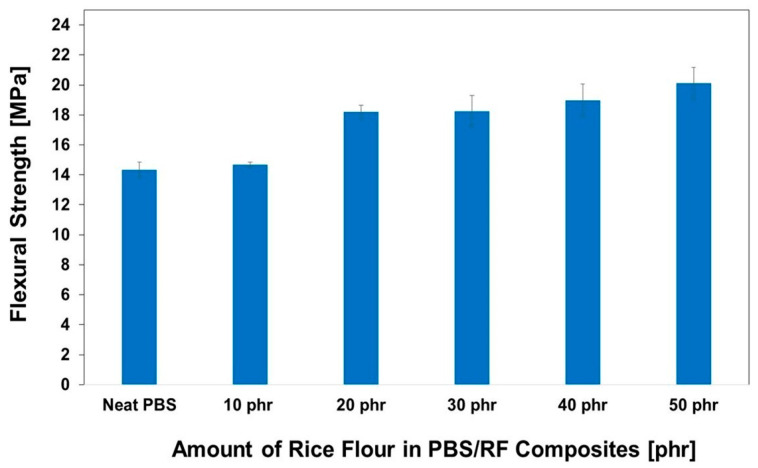
Flexural strength of PBS/RF biocomposites.

**Figure 9:**
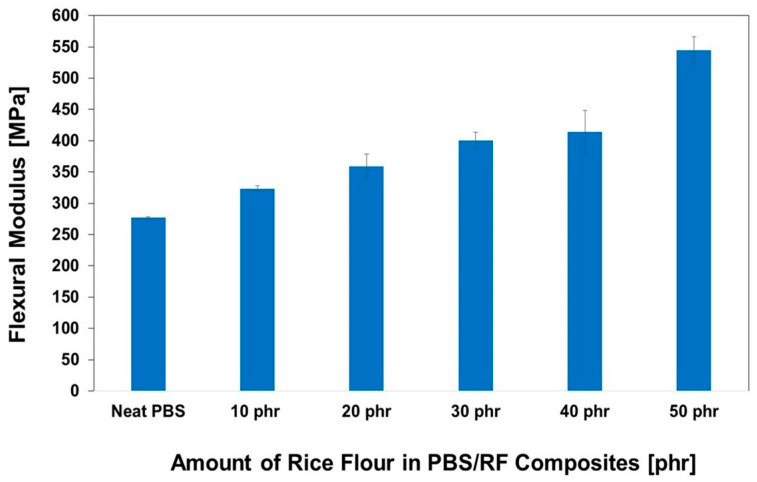
Flexural modulus of PBS/RF biocomposites.

The impact of RF filler content on the hardness of PBS/RF biocomposites was carefully investigated; the results are illustrated in Figure 7. Higher filler loading resulted in increased hardness due to the inherent stiffness of the filler. Incorporating RF filler at 10, 20, 30, 40, and 50 phr enhanced the hardness of the composites by 20.2%, 21.7%, 24.8%, 26.7%, and 27.3%, respectively, demonstrating the effectiveness of increasing filler content in improving composite surface properties. The hardness of the PBS/RF biocomposites increased with the addition of filler, which can be attributed to the incorporation of rigid particles that restrict surface deformation under an applied load. The presence of fillers hinders the segmental motion of polymer chains, effectively minimizing deformation under stress and contributing to the observed increase in hardness. These findings are consistent with those reported in previous studies [34,35]. Hammadi et al. (2023) reported that an increase in Al_2_O_3_ content leads to an increase in hardness. The addition of 10 wt.% Al_2_O_3_ resulted in an increase in hardness by up to 18.5% compared to the polymer matrix of the composite. This is because the added filler increases the rigidity of the material and its resistance to deformation [35]. Figure 8 and Figure 9 show the effect of the RF content on the flexural properties of the PBS/RF biocomposites. The presence of rigid filler particles at higher loads significantly improved the flexural strength and modulus of the PBS/RF biocomposites, suggesting their effectiveness in reinforcing the structure and restricting flexural motion. Adding 50 phr RF to the PBS matrix greatly improved both the flexural strength and flexural modulus, with increases of 40.3% and 96.5%, respectively. The incorporation of the RF filler into the PBS matrix resulted in a pronounced enhancement in both flexural strength and modulus. This improvement can be attributed to a synergistic reinforcement effect arising from more efficient stress transfer between the rigid filler and the PBS matrix, as well as a more uniform dispersion of the reinforcement phase, which is consistent with previous studies [6].

### 3.4. Soil Burial Degradation Test

**Figure 10:**
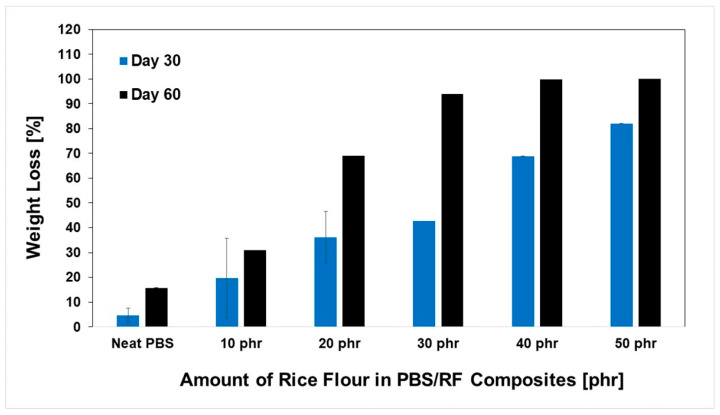
Weight loss (%) of PBS/RF biocomposites (film) during soil burial degradation tests.

**Figure 11:**
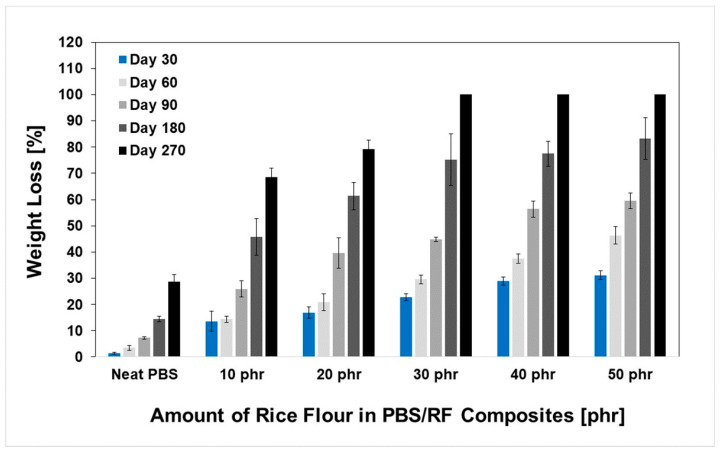
Weight loss (%) of PBS/RF biocomposites (specimen) during soil burial degradation tests.

The development of biocomposites by integrating a biodegradable filler (rice flour: RF) into the PBS matrix showed significant potential for producing materials with accelerated degradation profiles, which could be attributed to the biodegradability of both phases. Owing to its inherent biodegradability and moisture affinity, rice flour acts as a biological trigger that accelerates the breakdown of composite structures under natural conditions. The degradation rates of the PBS/RF biocomposites were determined with a soil burial degradation test at various times: 30, 60, 90, 180, and 270 days. Material weight loss was recorded, and the results are presented in Figure 9 and Figure 10. Soil burial degradation tests were conducted on both PBS/RF biocomposite films (10 mm × 130 mm × 0.03 mm) and specimens (1 cm × 5 cm × 0.3 cm) to determine the influence of RF loading, demonstrating weight loss in both the neat PBS and PBS/RF biocomposite materials. The degradation behavior of both specimens and films demonstrated a similar pattern, revealing a clear enhancement in degradation rate correlating with the incremental addition of filler ranging from 0 to 50 phr. Remarkably, the film samples achieved almost complete degradation (~100%) within 60 days (Figure 10), while the specimens required up to 270 days to reach a similar extent of degradation (Figure 11). The weight loss percentage of the PBS/RF biocomposites surpassed that of the neat PBS, highlighting the role of the RF filler in accelerating the biodegradation process.

Figure 11 shows the relationship between weight loss and the amount of filler in PBS/RF biocomposites (specimen) during soil burial degradation tests. After 30 days, the PBS/RF biocomposites (specimens) showed an increase in weight loss with an increase in the amount of RF filler, reaching 1.47%, 13.60%, 16.92%, 22.79%, 28.84%, and 31.09% for filler amounts of 0, 10, 20, 30, 40, and 50 phr, respectively. Within 270 days, the PBS/RF biocomposites containing 30 to 50 phr of RF filler exhibited nearly complete degradation under soil burial conditions, indicating their high susceptibility to biodegradation. It has been demonstrated that natural fillers facilitate the biodegradation of PBS during composting, and the extent of this enhancement strongly depends on the filler content [12]. Additionally, the soil burial degradation study results were consistent with the amount of water absorbed by the samples, confirming that as the RF filler content increased, the composites exhibited a greater extent of biodegradation, which can be attributed to the increased availability of water-accessible regions and the inherent degradability of the RF filler phase [1,2,5,6,12]. As rice particles degrade, they leave behind micro-voids or pores within the polymer matrix. These pores enhance water penetration, which promotes microbial colonization and deeper material degradation.

**Figure 12:**
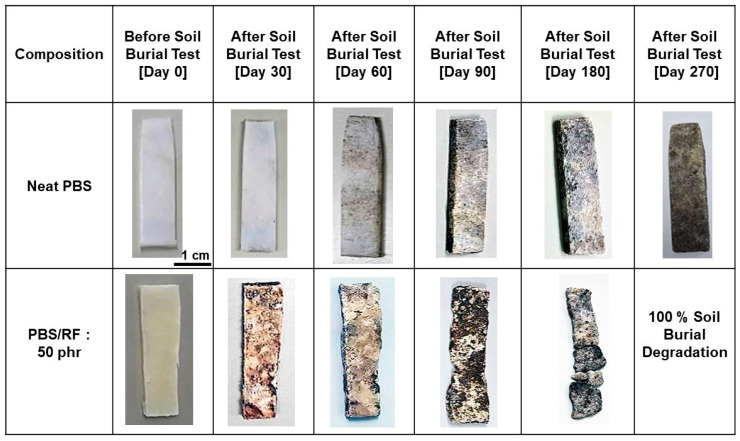
Visual changes in soil-buried PBS/RF biocomposite specimens (control (day 0) and specimens retrieved on days 30, 60, 90, 180, and 270).

The soil burial degradation of PBS was further confirmed by visually observing the physical appearance of neat PBS and PBS/50 phr RF biocomposite specimens after being buried in soil for various durations. After 30 days, the neat PBS samples showed little to no change in surface appearance, indicating a slower degradation rate compared to the PBS/RF biocomposites. The presence of natural fillers increases water absorption, which in turn promotes microbial growth and enzymatic activity, thereby accelerating the overall degradation of the composites in soil. According to the visual evidence presented in Figure 12, the PBS/RF biocomposite specimen with 50 phr of filler gradually decomposed and became nearly indistinct from the soil after a burial period of approximately 270 days. It is observed that after soil burial, the surface of the PBS/RF biocomposites becomes rough and exhibits visible cracks and pores, which facilitates the penetration of water and soil microorganisms. This observation underscores the potential of RF-filled biocomposites for sustainable applications. The results suggest that increasing the RF content enhances the environmental friendliness of the material and promotes degradation and integration into ecological systems. These observations concur with prior research, which also reported that PLA/NR/RS biocomposites show superior biodegradation performance compared to neat PLA and PLA/NR blends. Moreover, the degradation rate increased with the amount of rice straw natural filler because the hydrophilic nature of rice straw enhances water absorption, surface disintegration, and breakdown into small molecules [9].

**Figure 13:**
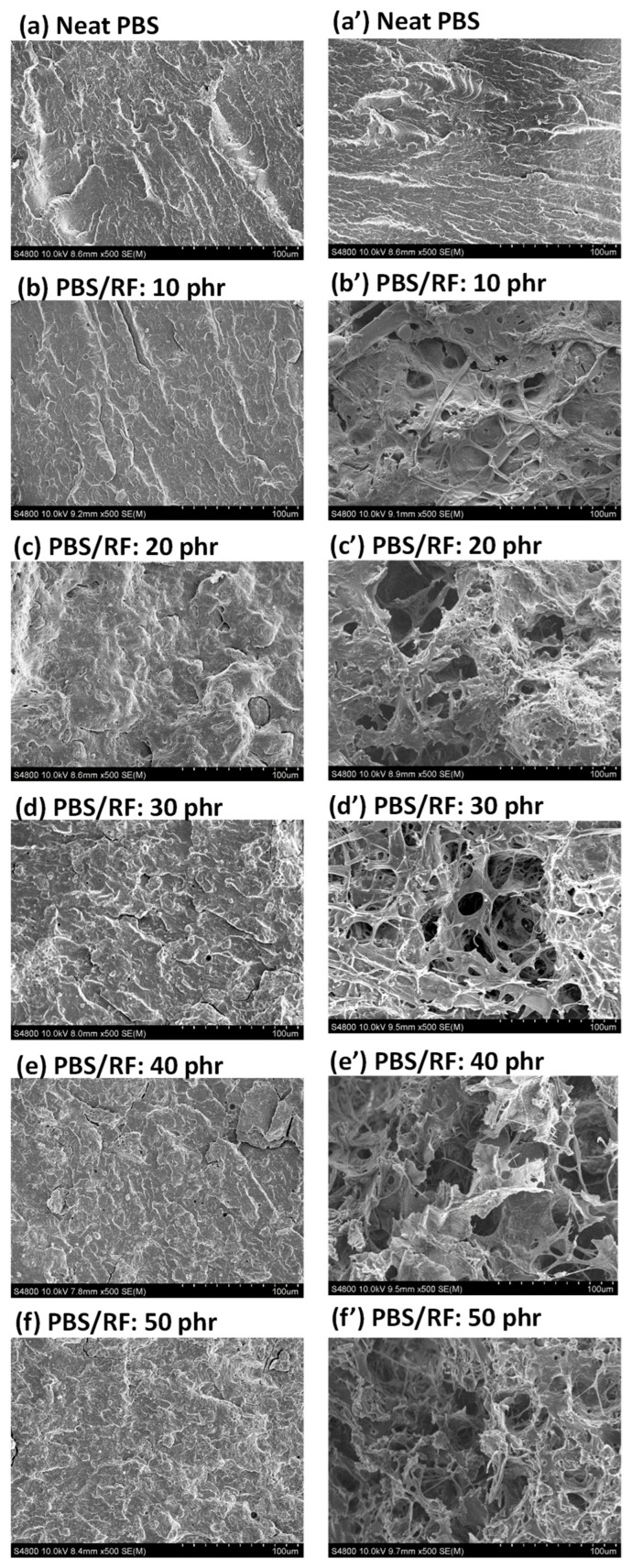
SEM images of PBS/RF biocomposites retrieved before and after soil burial experiments ((**a**–**f**): before soil burial test; (**a’**–**f’**): after soil burial test).

Rice flour is rich in starch, cellulose, and hemicellulose, all of which are readily biodegradable by microorganisms in natural environments. The incorporation of rice flour into PBS matrices promotes degradation by introducing hydrolysis-prone regions and enhancing microbial accessibility through filler-induced structural discontinuities. SEM images of the fracture surfaces of neat PBS and PBS/RF biocomposites after the soil burial test (day 180) are presented in Figure 13. Figure 13a–f illustrate the surface appearance of the neat PBS and PBS/RF composite specimens prior to soil burial, while Figure 13a’–f’ show the corresponding specimens after the soil burial degradation. The micrographs reveal distinct morphological differences; even after soil burial degradation, the neat PBS exhibited a smooth and uniform fracture surface, suggesting negligible morphological changes and a slower degradation rate. Conversely, the PBS/RF biocomposites exhibited rougher surfaces and the presence of voids, suggesting that degradation occurs more easily according to the RF filler. Moreover, the degradation was more extensive at higher filler loadings. This finding indicates that the RF filler not only influences the mechanical properties of the biocomposites but also accelerates the degradation process when subjected to environmental conditions. This enhancement of degradation through natural filler incorporation aligns with the observations of Kim et al. (2006), who reported that bio-flour-filled PBS composites exhibited a higher degradation rate compared with the neat polymer [36].

## 4. Conclusions

The incorporation of bio-based components resulted in improved mechanical performance while simultaneously enabling rapid biodegradation, making them excellent options for eco-friendly uses. Polybutylene succinate (PBS)/rice flour (RF) biocomposites with an RF amount of 0, 10, 20, 30, 40, and 50 phr were successfully fabricated and investigated. The results demonstrated that PBS/RF biocomposites exhibited enhanced mechanical properties and underwent accelerated biodegradation, highlighting their potential as eco-efficient materials for sustainable applications. The incorporation of 50 phr of RF filler led to significant enhancements in the mechanical properties of the PBS matrix, with increases of 257%, 40.3%, 96.5%, and 27% in Young’s modulus, flexural strength, flexural modulus, and hardness, respectively. Moreover, the PBS/RF biocomposites exhibited a maximum water absorption capacity of 22% and showed accelerated degradation behavior, with weight loss reaching 100% after 270 days of soil burial, indicating improved environmental degradability. This remarkable performance demonstrates the strength of PBS/RF biocomposites in reducing plastic waste and promoting sustainability.

## Figures and Tables

**Table 1:** Composition of different PBS/RF biocomposites.

Materials for PBS/RF Biocomposites		
RF treated with silane [phr]	ENR-50 [phr]	Code
0	0	Neat PBS
10	1	PBS/RF: 10 phr
20	1	PBS/RF: 20 phr
30	1	PBS/RF: 30 phr
40	1	PBS/RF: 40 phr
50	1	PBS/RF: 50 phr

## Data Availability

The authors confirm that the data supporting this study are available within the article. Raw data that support the findings of this study are available from the corresponding author upon reasonable request.
